# Infrapatellar Fat Pad Gene Expression and Protein Production in Patients with and without Osteoarthritis

**DOI:** 10.3390/ijms21176016

**Published:** 2020-08-21

**Authors:** Elisa Belluzzi, Veronica Macchi, Chiara Giulia Fontanella, Emanuele Luigi Carniel, Eleonora Olivotto, Giuseppe Filardo, Gloria Sarasin, Andrea Porzionato, Marnie Granzotto, Assunta Pozzuoli, Antonio Berizzi, Manuela Scioni, Raffaele De Caro, Pietro Ruggieri, Roberto Vettor, Roberta Ramonda, Marco Rossato, Marta Favero

**Affiliations:** 1 Musculoskeletal Pathology and Oncology Laboratory, Orthopedic and Traumatologic Clinic, Department of Surgery, Oncology and Gastroenterology (DISCOG), University of Padova, 35128 Padova, Italy; elisa.belluzzi@gmail.com (E.B.); assunta.pozzuoli@unipd.it (A.P.); 2 Institute of Human Anatomy, Department of Neurosciences, University of Padova, 35121 Padova, Italy; veronica.macchi@unipd.it (V.M.); gloria.sarasin@unipd.it (G.S.); andrea.porzionato@unipd.it (A.P.); raffaele.decaro@unipd.it (R.D.C.); 3 L.i.f.e. L.a.b. Program, Consorzio per la Ricerca Sanitaria (CORIS), Veneto Region, 35128 Padova, Italy; 4 Department of Civil, Environmental and Architectural Engineering, University of Padova, 35131 Padova, Italy; chiaragiulia.fontanella@unipd.it; 5 Centre for Mechanics of Biological Materials, University of Padova, 35131 Padova, Italy; emanueleluigi.carniel@unipd.it; 6 Department of Industrial Engineering, University of Padova, 35131 Padova, Italy; 7 RAMSES Laboratory, RIT Department, IRCCS Istituto Ortopedico Rizzoli, 40136 Bologna, Italy; eleonora.olivotto@ior.it; 8 Applied and Translational Research (ATR) Center, IRCCS Istituto Ortopedico Rizzoli, 40136 Bologna, Italy; g.filardo@biomec.ior.it; 9 Clinica Medica 3, Department of Medicine—DIMED, University of Padova, School of Medicine, 35128 Padova, Italy; marnie.granzotto@unipd.it (M.G.); roberto.vettor@unipd.it (R.V.); 10 Orthopaedic and Traumatologic Clinic, Department of Surgery, Oncology and Gastroenterology (DiSCOG), University of Padova, 35128 Padova, Italy; antonio.berizzi@unipd.it (A.B.); pietro.ruggieri@unipd.it (P.R.); 11 Department of Statistical Sciences, University of Padova, 35121 Padova, Italy; scioni@stat.unipd.it; 12 Rheumatology Unit, Department of Medicine-DIMED, University—Hospital of Padova, Via Giustiniani, 2, 35128 Padova, Italy; roberta.ramonda@unipd.it (R.R.); faveromarta@gmail.com (M.F.); 13 Internal Medicine I, Cà Foncello Hospital, 31100 Treviso, Italy

**Keywords:** adipocyte, infrapatellar fat pad, osteoarthritis, anterior cruciate ligament, inflammation

## Abstract

Osteoarthritis (OA) is one of the most common joint disorders. Evidence suggests that the infrapatellar fat pad (IFP) is directly involved in OA pathology. However, a comparison between OA versus non-OA IFP is still missing. Thus, the aim of this study was to compare IFP molecular, adipocytes and extracellular matrix characteristics of patients affected by OA, and patients undergoing anterior cruciate ligament (ACL) reconstruction. We hypothesized that not only inflammation but also changes in adipocytes and extracellular matrix (ECM) composition might be involved in OA pathogenesis. Fifty-three patients were enrolled. IFP biopsies were obtained, evaluating: (a) lymphocytic infiltration and vascularization; (b) adipocytes area and number; (c) adipo-cytokines and extracellular matrix gene expression levels; (d) IL-6 and VEGF protein production; (e) collagen fibers distribution. OA IFP was more inflamed and vascularized compared to ACL IFP. OA IFP adipocytes were larger and numerically lower (1.3-fold) than ACL IFP adipocytes. An increase of gene expression of typical white adipose tissue genes was observed in OA compared to ACL IFP. Collagen-types distribution was different in the OA IFP group compared to controls, possibly explaining the change of the biomechanical characteristics found in OA IFP. Statistical linear models revealed that the adipocyte area correlated with BMI in the OA group. In conclusion, inflammation and fibrotic changes of OA IFP could represent novel therapeutic targets to counteract OA.

## 1. Introduction

Osteoarthritis (OA) is one of the most frequent forms of arthritis and an important cause of pain and disability in elderly people [1]. The most affected joint is the knee, with a worldwide estimated radiographic prevalence of 3.8% [2].

Nowadays, OA is considered a whole joint disease involving cartilage, meniscus, synovial membrane, and infrapatellar fat pad (IFP) [3,4,5,6]. It is well-known that OA is characterized by synovial inflammation, determined by synoviocytes, which secrete pro-inflammatory cytokines that induce chondrocytes to produce degradative enzymes of the extracellular matrix (ECM) and inhibit both tissue repair and regeneration [7]. Actually, there is no cure for this pathology and the OA management relies on symptomatic interventions. Total joint replacement represents the only treatment available for end-stage OA. However, physical activity and nutrition supplements are considered as nonpharmacological and preventive treatment for OA and sarcopenia [7,8,9].

Recently, attention has focused on the role of the IFP in OA pathophysiology [3,10,11]. It has been shown that IFP produces pro-inflammatory mediators inducing synovial inflammation and seems to act as an anatomo-functional unit with synovial membrane contributing to OA onset and progression [3,10,11,12,13,14].

Moreover, during the last years, research has focused on the study of IFP-derived stem cells for regenerative medicine [15]. Recently, we showed that OA-IFP stem cells seem to be primed by the pathological environment and to exert incomplete protective activity from OA inflammation [16].

A decrease of IFP volume and an increase of hypointense signal at the magnetic resonance imaging (hallmarks of fibrosis) were described in OA patients compared to controls [17]. It has been reported that IFP signal intensity alterations were associated with the incidence of radiographic OA [18] and that IFP hypointense signals were associated with increased knee cartilage defects and bone marrow lesions [19]. Moreover, IFP undergoes biomechanical changes in OA, showing a nonorganized distribution of the stresses within the interlobular septa affecting the mechanical (and possibly functional) response of the adipose lobules [20]. Usually, gene expression of OA IFP is compared with that of subcutaneous adipose tissue of the same subject, although these depots are very different. In this regard, the comparison of different IFP conditions could allow us to better understand and quantify the specific disease-related changes of the IFP.

The aim of the present study was to compare the histological, morphometric, and molecular characteristics of IFP of patients undergoing total knee replacement (TKR) for end-stage OA with those of patients undergoing anterior cruciate ligament reconstruction (ACLR) after traumatic rupture. We hypothesized that not only inflammation but also changes in adipocytes and extracellular matrix (ECM) composition might be involved in OA pathogenesis.

## 2. Results

### 2.1. Demographic and Clinical Characteristics of Patients

Twenty-eight patients undergoing ACLR for traumatic rupture and twenty-five patients undergoing TKR for end-stage OA were enrolled. Patients’ characteristics are summarized in Table S2, Supplementary Materials. The time between the injury and the surgery of patients with ACLR was at least 6 months (median 8 months; interquartile range (IQR), 14.5–6). Males were 75% in the ACL group and 28% in the OA group (*p* = 0.001). Moreover, ACL subjects were statistically younger (median age 31; IQR, 42–22) compared to OA patients (median age 68; IQR, 75–62) (*p* < 0.0001). The BMI of the ACL group (median BMI 23.04; IQR, 25.26–20.57) was statistically lower than that of OA patients (median 29.52; IQR, 32.25–25.95) (*p* < 0.0001).

### 2.2. Histopathological Grading

The IFP histopathological grading was evaluated in 23 ACL IFP and 25 OA IFP (Table 1). It was not possible to perform the histological analysis in 3 ACL samples due to the small volume of the biopsies. Lymphocytic infiltration was substantially absent in all ACL IFP (grade 0) except for one, which was graded as 1. On the contrary, lymphocytic infiltration was present in the majority of OA IFP: 28% of the OA IFP was graded as 1 and 52% as 2. Lymphocytic infiltration was found to be statistically different between the two groups (*p* < 0.0001). Vascularity was increased in OA IFP (median 30.6; 42.9–30.6) compared to ACL IFP (median 8.9; 17.5–7.2) (*p* < 0.0001).

### 2.3. Adipocyte Morphology Evaluation

**Figure 1:**
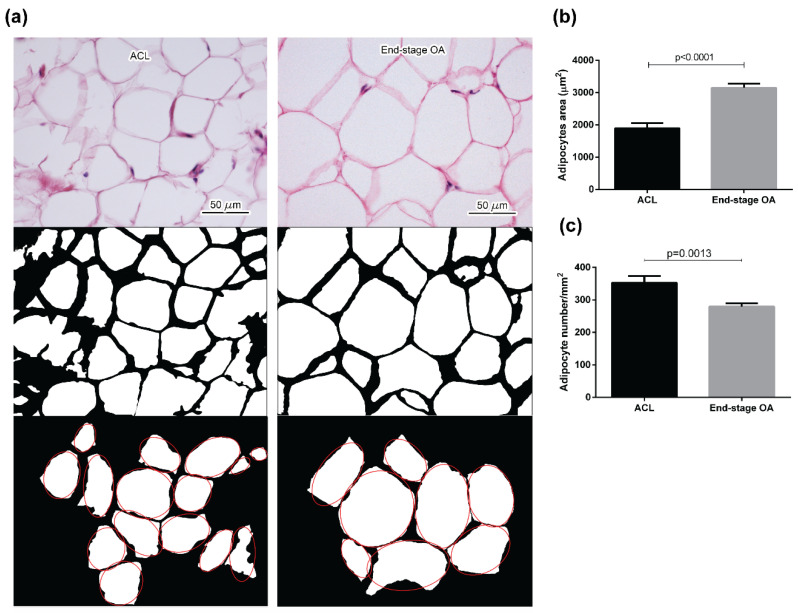
Adipocytes morphology evaluation. (**a**) ACL (on the left) and end-stage OA (on the right) adipocytes were reported from HE stained sections and then converted into the corresponding binary images. For each image, adipocytes areas (white regions) were identified and approximated to ellipses (red line). The comparison between adipocytes area (**b**) and number (**c**) for ACL and end-stage OA (Median and IQR) showed the increment of adipocytes size and the decrease of adipocytes number in OA IFP. ACL = anterior cruciate ligament; OA = osteoarthritis.

Adipocytes dimension in ACL and OA IFP was quantified, showing an increase of the cell area in adipocytes of OA IFP compared to ACL IFP (*p* < 0.0001) (Figure 1a,b and Table 2). The number of adipocytes was evaluated, revealing a decrease in OA compared to ACL IFP (*p* = 0.0013) consistent with the increase of the adipocyte area in OA (Figure 1c).

### 2.4. Gene Expression Analysis and Immunohistochemistry

#### 2.4.1. Inflammation and Vascularization

In order to compare cytokines and chemokines gene expression of ACL IFP to OA IFP, qRT-PCR was performed in eight samples for each group (Figures S1 and S2 Supplementary Materials).

The median age and BMI were statistically different between the two subgroups as well as in the whole population (*p* < 0.0001 and *p* = 0.010, respectively).

**Figure 2:**
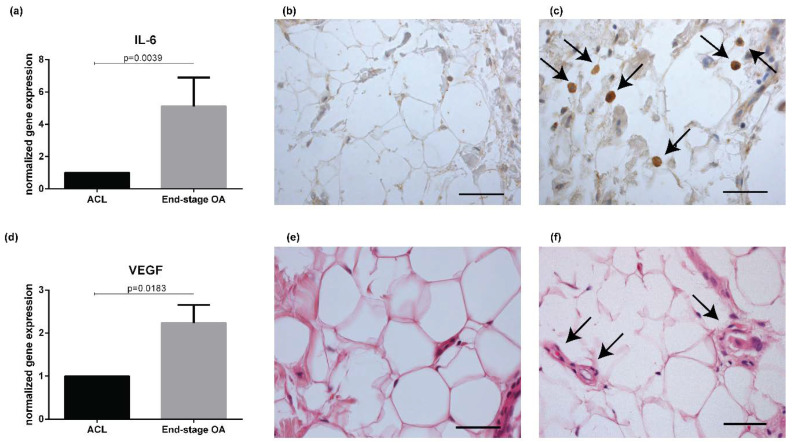
Evaluation of inflammation and vascularization in IFP of ACL and end-stage OA patients. *IL-6* gene expression revealed an increase of *IL-6* expression in end-stage OA IFP (*n* = 8) compared to ACL IFP (*n* = 8) (**a**). Immunohistochemistry of IL-6 end-stage OF IFP showed a marked positivity (highlighted by the arrows) (**c**) compared to ACL IFP (**b**). *VEGF* gene expression levels were increased in end-stage OA (*n* = 8) compared to ACL IFP (*n* = 8) (**d**). Hematoxylin–eosin image showed an increased in end-stage OA IFP (highlighted by the arrows) (**f**) compared to ACL IFP (**e**). Scale bar b = 50 μm; c = 23.8 μm; e,f = 37.5 μm. IL = interleukin; ACL = anterior cruciate ligament; IFP = infrapatellar fat pad; OA = osteoarthritis; VEGF = vascular endothelial grow factor.

A statistical difference was observed between the two subgroups in *IL-6* and *VEGF* gene expression levels (*p* = 0.0039 and *p* = 0.0183, respectively) (Figure 2a–d), while no differences were observed in *MCP-1/CCL-2* and *TNF-α* expression (Figure S1, Supplementary Materials). A decrease in *TGF-β* gene expression levels was determined in OA IFP compared to ACL IFP (*p* = 0.0368) (Figure S1, Supplementary Materials). On the basis of these results, IL-6 and VEGF proteins were evaluated by IHC or HE in a group of patients confirming the difference detected by gene expression between ACL (*n* = 21) and OA IFP (*n* = 14) (Figure 2b,c,e,f) (Table S3, Supplementary Materials).

#### 2.4.2. Adipokines

**Figure 3:**
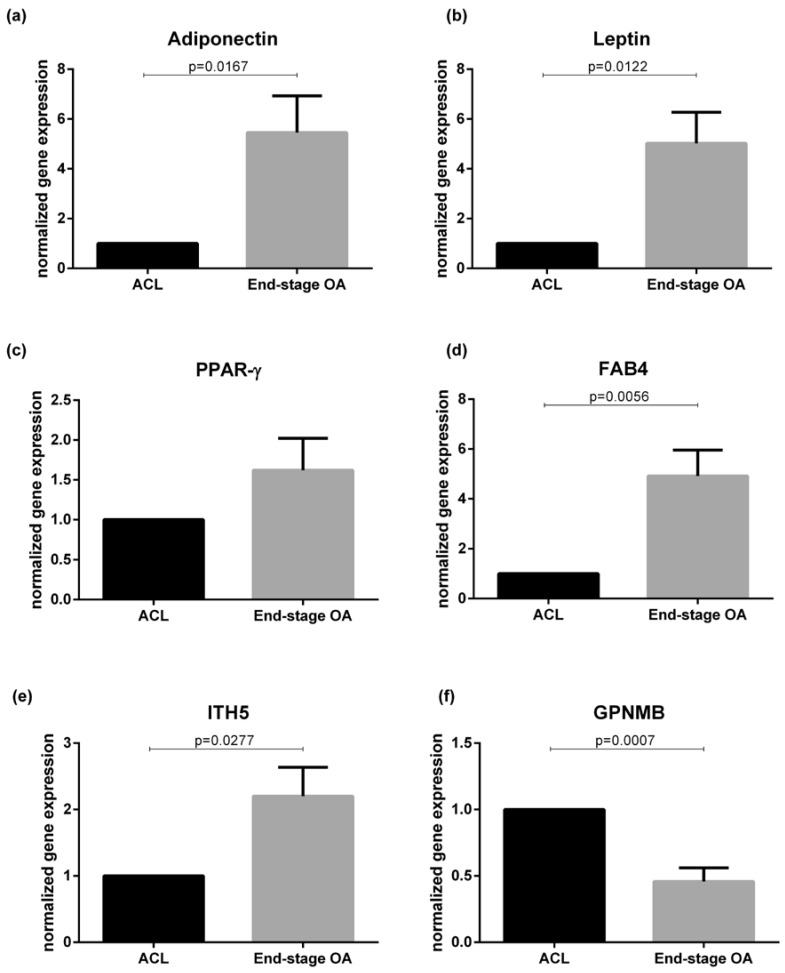
Adipokines evaluation in IFP ACL (*n* = 8) and end-stage OA patients (*n* = 8). (**a**) *Adiponectin* gene expression levels were increased in end-stage OA compared to ACL IFP. (**b**) *Leptin* gene expression levels were increased in end-stage OA compared to ACL IFP. (**c**) *PPAR-γ* gene expression levels were increased in end-stage OA compared to ACL IFP. (**d**) *FAB4* gene expression levels were increased in end-stage OA compared to ACL IFP. (**e**) *ITH5* gene expression was higher in end-stage OA IFP. (**f**) *GPNMB* gene expression was higher in ACL IFP. IFP = infrapatellar fat pad; ACL = anterior cruciate ligament; OA = osteoarthritis; PPAR-γ = peroxisome proliferative activated receptor gamma; FABP4 = fatty acid-binding protein 4; ITH5 = inter-α-trypsin inhibitor heavy chain 5; GPNMB = transmembrane glycoprotein NMB.

Gene expression analysis of different adipokines was performed comparing ACL and OA IFP samples (Figure 3). There was an increase of white adipose tissue typical genes (*adiponectin*, *leptin,* and *FAB4*) in end-stage OA compared to ACL IFP (*p* = 0.0167, *p* = 0.0122, and *p* = 0.0056, respectively), while no differences were observed regarding *PPAR-*γ (*p* = 0.1590). The expression of *GPMNB* was evaluated, showing a decrease in end-stage OA compared to ACL IFP (*p* = 0.0007). On the contrary, an increase of *ITH5* gene expression was shown in end-stage OA compared to ACL IFP (*p* = 0.0277). No differences were evident in *SERPIN2* gene expression between the two groups (Figure S1, Supplementary Materials).

#### 2.4.3. Extracellular Matrix Remodeling

**Figure 4:**
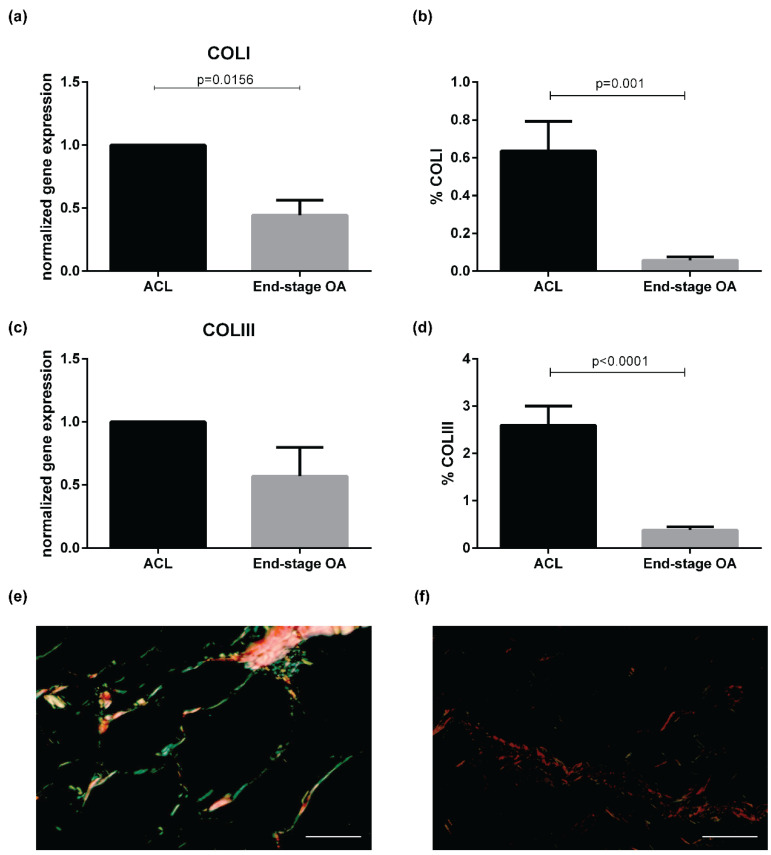
Extracellular matrix remodeling gene expression in ACL IFP (*n* = 8) and end-stage OA IFP (*n* = 8) and Sirius red evaluation. (**a**) *COLI* gene expression was higher in ACL IFP compared to end-stage OA. (**b**) COLI was higher in ACL IFP compared to end-stage OA evaluated by Sirius red. (**c**) *COLIII* gene expression was higher in ACL IFP compared to end-stage OA. (**d**) COLIII was higher in ACL IFP compared to end-stage OA evaluated by Sirius red. (**e**) Sirius red staining of ACL IFP (COLI (thick fibers) = yellow-red birefringence; COLIII (thin fibers = green birefringence). (**f**) Sirius red staining of end-stage OA IFP (COLI (thick fibers) = yellow-red birefringence; COLIII (Thin fibers = green birefringence). Scale bars = 50 μm. COLI = collagen type I; COLIII = collagen type 3; ACL = anterior cruciate ligament; IFP = infrapatellar fat pad; OA = osteoarthritis.

The analysis of the expression of genes involved in ECM remodeling revealed a decrease of *COLI* expression in end-stage OA compared to ACL IFP (*p* = 0.0156) (Figure 4a), while no differences were detected in both *COLIII* (Figure 4c) and *COLVI* (Figure S2, Supplementary Materials) gene expression (*p* = 0.1289 and *p* = 0.598, respectively). COLI and COLIII were evaluated also by Sirius red (Figure 4) showing a decrease of both collagen proteins in OA (*n* = 14) compared to ACL IFP (*n* = 18) (*p* = 0.001, *p* < 0.0001, respectively).

### 2.5. Correlations Between Histological Data and Morphometric Analysis

The age of overall cohort correlated positively with the adipocyte area (r = 0.591, *p* < 0.0001), and negatively with the adipocyte number (r = −0.412, *p* = 0.003) (Figure S3a,b, Supplementary Materials). However, considering the two groups separately, both correlations were not maintained. The adipocytes area was positively correlated with BMI of overall cohort (r = 0.621, *p* < 0.0001) (Figure S3c, Supplementary Materials) while, separating the two groups, this correlation was present only in end-stage OA IFP (r = 0.413, *p* = 0.040) (Figure S3e,f, Supplementary Materials). The number of adipocytes of the overall cohort was negatively correlated with BMI (r = −0.427, *p* = 0.003) (Figure S3d, Supplementary Materials) and this correlation was maintained only in the OA group (r = −0.514, *p* = 0.009) (Figure S3g,h, Supplementary Materials).

### 2.6. Influence of BMI and Age

Since well-known risk factors for OA, such as BMI and age, were not equally distributed between the two patient groups, we applied linear models to control their influence (Tables S4–S6, Supplementary Materials). Vascularity did not fit a normal distribution, and so it was log-transformed before being modeled. Lymphocytic infiltration, IL-6 immunohistochemical grading, vascularity, and adipocyte numbers were all independent from BMI and age. Furthermore, BMI and age did not have any significant effect on the response variables, whether analyzing OA and ACL rupture separately or combined. Only the adipocyte area was almost associated with BMI in the OA group (*p* = 0.057) and significantly associated when the two patient groups were considered together (*p* = 0.033).

## 3. Discussion

This is the first study investigating the histological, morphometric, and molecular characteristics of end-stage OA IFP compared to ACL IFP. In particular, we have explored not only IFP inflammation but also adipocytes and ECM changes in these two groups. The studies published so far mostly focalized on IFP inflammation and utilized the subcutaneous adipose tissue of the knee region as “healthy” control, which might not be an optimal “healthy” control because of the differences in physiological functions and biomechanical characteristics of these fat depots [21]. Furthermore, OA IFP characterization is of great importance to unravel its role in OA pathology as occurred for other tissues such as synovial membrane and cartilage [22,23].

The important novelty of this study is the evidence that, beyond inflammation, also adipocytes and ECM characteristics of OA patients are different compared to IFP derived from ACL patients. In particular, these data suggest that OA pathology induces molecular changes in IFP, affecting the cells and ECM composition in addition to the increase of inflammation.

Regarding the adipocyte characteristics, we observed that the adipocyte area was 1.7-fold higher in end-stage OA compared to ACL IFP, while the adipocyte number was lower in end-stage OA compared to ACL IFP. Interestingly, the adipocyte area of IFP was positively correlated with BMI, while adipocyte number was negatively correlated with BMI, but only the adipocyte area was confirmed to be associated with the BMI at linear regression model in the overall population and in the end-stage OA group. The absence of the correlation in the ACL IFP group between BMI and adipocyte area could be explained, considering that all these subjects were lean.

Previous studies evaluated the adipocyte area in OA IFP subgrouping the patients according to BMI, observing that the area was smaller in lean OA patients (BMI < 25 kg/m^2^) compared to severely obese OA patients (BMI ≥ 35 kg/m^2^) [24]. In contrast, de Jon et al. did not observe any difference in adipocyte size of OA IFP related to patient BMI, reporting that adipocyte volume and size of OA IFP were smaller compared to subcutaneous adipose tissue used as control [25]. The influence of BMI on the adipocyte area was not confirmed in other studies, both in animals and humans. Barboza et al. showed an increase of IFP volume in mice with high-fat diet-induced obesity compared to controls. In contrast, no difference was reported in the adipocyte area, suggesting that obesity does not influence this IFP feature [26]. In addition, other authors demonstrated that BMI did not exhibit any effect on adipocyte area in humans [27].

In general, the adipocyte area has been studied from all adipose tissue anatomical locations and in both sexes. The adipocyte area increases in size along with adiposity level, reaching a plateau in massively obese subjects [28]. Here, we showed that age was positively associated with the adipocyte area and negatively with the adipocyte number of IFP, although these findings were not confirmed by the linear regression model. No specific studies have been published so far evaluating the effects of age on IFP adipocyte characteristics. However, no significant association was observed between age and adipocyte size distribution parameters in omental and subcutaneous adipose tissue [29].

We observed an increase of lymphocytic infiltration as well as of vascularity in end-stage OA IFP compared to ACL IFP, confirming our previous data obtained comparing end-stage OA IFP with that of cadavers considered as healthy controls [30]. Increased lymphocytic infiltration and vascularity were independent of age and BMI in both groups in the linear models, suggesting that these inflammatory features play an important role in OA pathology. Moreover, in agreement with the histological IFP scores, we observed an increase of *IL-6* and *VEGF* mRNA expression levels in OA compared to ACL IFP. The increase of IL-6 expression was also confirmed by IHC in agreement with our previous results [30]. Surprisingly, we did not observe increased MCP-1 expression levels in OA IFP compared to ACL IFP, at variance with what we observed in a previous study using IHC [30]. The expression of all classical adipose tissue markers was increased in OA IFP compared to ACL IFP, in agreement with previously published data showing an increase of adipogenesis genes in end-stage OA IFP compared to early OA IFP [31].

The expression of other genes putatively involved in adipose tissue ECM organization and cell differentiation, such as *SERPIN2, GPNMB,* and *ITH5* considered as novel adipokines, was investigated only in OA IFP patients without any comparison with controls [32]. Differences in *GPNMB* and *ITH5* expression were observed between the OA and ACL IFP, with no differences for *SERPIN2*. Several studies highlighted the presence of fibrosis in OA IFP compared to non-OA tissues and subcutaneous adipose tissue [13,30]. GPNMB is a transmembrane protein involved in adipose tissue-derived inflammation in a mouse model of obesity [33]. We showed an increase of *GPNMB* in OA compared with ACL IFP with the increase of the inflammatory pattern in OA IFP. ITIs proteins are directly involved in the stabilization of ECM forming complexes binding hyaluronic acid molecules [34]. ITIH5 is highly expressed in white adipose tissue, and the expression seems to be increased in obese subjects [35]. Here, we observed an increase in its expression in OA IFP, in agreement with the increase of fibrosis. However, we cannot exclude that the differences observed in GPNMB and ITIH5 expression could be influenced by BMI, an important risk factor for OA.

The expression of other genes involved in the ECM composition was also investigated. ECM of adipose tissue is composed of several types of collagen and is particularly rich in COLVI that is positively correlated with BMI [36]. We evaluated *COLI, III,* and *COLVI,* showing that *COLI* and *COLIII* levels were decreased in OA compared to ACL IFP, while no differences were detected in *COLVI* expression. The differences in COLI and COLIII were also confirmed at the protein level, supporting the hypothesis of a change in ECM composition in OA, leading to the fibrotic phenotype of OA IFP [24,30]. This could also explain the reason why OA IFP has a biomechanical behavior different from that of healthy IFP [20].

Our study pointed out for the first time that OA pathology has a direct impact on IFP ECM and, in particular, on the expression of collagens and adipokines involved in the fibrotic process. These findings might open the possibility that fibrosis could be the target of a novel therapeutic strategy to counteract the OA progression and related pain in OA patients. This is also supported by a recently published paper by Onuma et al. that established a novel inflammation-induced knee pain model in rats and showed that the inflammation-induced fibrotic changes in the IFP were associated with the prolongation of joint pain [37]. The main limitation of our work is the age and BMI differences between the two groups of subjects, due to their specific categorization, given the fact that ACL rupture usually occurs in young, lean athletes, while OA occurs mainly in aging females. However, we showed that our analysis was not influenced by these confounders by general linear models except for the adipocyte area in the OA group and the overall cohort. In particular, inflammatory features such as lymphocytic infiltration, vascularization, and IL-6 protein expression as evaluated by IHC were increased in OA IFP compared to controls independently from BMI, suggesting an important role of IFP inflammation in OA pathogenesis. Unfortunately, it was not possible to apply the linear models to the gene expression analysis due to the small sample size. In addition, we cannot exclude that the acute trauma occurring during ACL rupture influenced our analysis [38]. However, in our study, we have enrolled patients who underwent ACLR at least 6 months after the injury in order to avoid the inflammatory phase occurring after the trauma. Bigoni et al. analyzed cytokines levels in synovial fluid of patients with ACL tears divided in the study population into 4 groups according to the time elapsed from the injury. Those authors demonstrated that there was an increased level of pro-inflammatory cytokines in the acute phase of inflammation, followed by a decrease with a minimum of three months after injury [39]. Current studies on patients with ACLR are mainly focused on the evaluation of inflammatory markers in the synovial fluid. Heilmeier et al. correlated the synovial fluid inflammatory markers with the IFP/synovial membrane abnormalities detected by MRI [40]. They found that the degree of IFP abnormality correlated positively with the synovial fluid levels of the inflammatory cytokine markers and with chondro-destructive markers as MMP-1 and −3. Since these Authors did not analyze IFP and synovial membrane inflammatory cytokines expression, it is not possible to discern the contribution of each joint tissue in the secretion of these inflammatory markers [40].

## 4. Materials and Methods

### 4.1. Study Population

Patients undergoing ACLR were enrolled at the IRCSS Rizzoli Orthopedic Institute (Bologna, Italy), while patients undergoing TKR for end-stage OA were enrolled at the Orthopaedic Clinic (University-Hospital of Padova, Padova, Italy). The Local Ethical Committees approved the study protocol and all patients signed written informed consent (approval code: 4510/AO/18, approval date: 18 July 2019). Patients or the public were not involved in the design, or conduct, or report, or disseminate plans of our research. Patients with previous knee surgery or other significant pathologies were excluded from the study. For each patient, demographic and clinical data were recorded.

Small biopsies of IFP were obtained during knee arthroscopy for ACLR or TKR surgery.

### 4.2. Histology and Immunohistochemistry

From the paraffin-embedded samples, 10 μm thick sections were obtained and stained with hematoxylin–eosin (HE) and Sirius red. For each case the IFP’s score was applied considering the presence of lymphocytic infiltration and vascularization as published [21]. The adipose lobules dimension and the thickness of the interlobular septa were not evaluated due to the small volume of the IFP biopsies obtained from the ACL patients and gene expression analysis was not performed in all patients of this group.

All sections were analyzed under a DM4500-B light microscope (Leica Microsystems, Wetzlar, Germany) and recorded in full color (24 bit) with a digital camera (DFC 480, Leica Microsystems).

Collagen subtypes were studied on sections stained with Sirius red and quantified as previously described [41].

For immunohistochemical (IHC) analysis, an anti-IL-6 antibody (polyclonal mouse antibody, Thermo Fisher) was used at 1:200 without antigen retrieval. Tissue sections were incubated using the DAKO Autostainer System (EnVision™ FLEX, High pH). The presence of positive cells was evaluated and graded as follows: grade 0 = absence of positive cells, grade 1 = weak positive cells, grade 1 = rare strong positive cells, grade 2 = clustered strong positive cells, grade 3 = diffuse strong positive cells.

### 4.3. Morphometric Analysis

After digitizing the images acquired at 10× magnification from HE stained sections, images were converted to binary images (black-white) for the elaboration with a Programming Language Software (Matlab R2018b, The MathWorks, Inc., Natick, MA, USA). A specific procedure was adopted to identify the boundary of the adipocytes, considering the connected regions of similar intensity in the grayscale images [20,30,41]. The white regions represent the adipocytes and the black region, the boundary.

In each image, adipocytes were approximated to ellipses, and areas and major/minor axes were calculated. A count of the number of adipocytes for each image was calculated.

### 4.4. Gene Expression Analysis

Total RNA was extracted from IFP away from the synovial membrane using QIAMP mini kit (Qiagen, Hilden, Germany) following the manufacturer’s protocol. First-strand cDNAs were synthesized from equal amounts of total RNA using random primers and M-MLV reverse transcriptase (Promega, Madison, WI, USA). Quantitative real-time PCR (qRT-PCR) for adipokines (leptin, adiponectin, peroxisome proliferative activated receptor gamma2 [PPARγ], fatty acid binding protein 4 [FABP4]), cytokines (IL-6, TNF-α, monocyte chemotactic protein 1 [MCP-1]), vascular endothelial growth factor (VEGF), chemokines (C-X-C motif chemokine ligand 8 [CXCL8]), genes involved in extracellular matrix remodeling (collagen type I [COLI], collagen type III [COLIII], and collagen type VI [COLVI], transmembrane glycoprotein NMB [GPNMB], inter-α-trypsin inhibitor heavy chain 5 [ITH5], serine proteinase inhibitor 2 [SERPIN2]), and transforming growth factor (TGF-β) was performed using Sybr-Green fluorophore and specific primers (Table S1, Supplementary Materials). Reaction efficiency was established for each set of primers and for an endogenous unregulated reference gene (18 s), after quantification of six different dilutions of the cDNA pool and calculated from the slope according to the equation E = 10^−1^/slope [42]. qRT-PCR was performed in triplicate for each gene and carried out in duplicate for each sample by DNA Engine Opticon 2 (MJ Research, Waltham, MA, USA). Melting curves confirmed the specificity of the amplification signal target of our gene. Data were calculated using the comparative (ΔΔCt) method as the ratio of each gene to the expression of the housekeeping gene and are represented as fold-change versus controls.

### 4.5. Statistical Analysis

The Shapiro–Wilk’s test was used to determine whether data were distributed normally. Chi-square (χ^2^) test or Fisher’s exact test were performed to compare categorical and dichotomous data. Mann–Whitney test or unpaired *t*-test were used to compare continuous variables. Spearman’s or Pearson’s correlations were performed to analyze associations between continuous variables. One-way ANOVA or Kruskal–Wallis, with Tukey’s post-hoc test, were used to analyze categorical data. Continuous variables were reported as median ± interquartile range (IQR), while categorical variables were reported as frequency and percentage. Generalized linear regression models were applied to investigate the association of the ACL and OA IFP variables with BMI and age. A *p* < 0.05 was considered as statistically significant. All analyses were performed with SPSS version 23.0 or R [43].

## 5. Conclusions

In conclusion, we confirmed that OA IFP is more inflamed and vascularized compared to ACL IFP, both at the histological and molecular levels. Moreover, we found that OA IFP adipocytes are larger and numerically lower than ACL IFP adipocytes. ECM collagen types distribution in the OA IFP group is different compared to controls, possibly explaining the biomechanical characteristics changes of OA IFP tissue. This study supports the hypothesis that IFP is involved in OA pathology. The clinical relevance of this work is that inflammation and fibrosis of IFP could represent possible therapeutic targets to counteract OA pathology. In this regard, future studies targeting inflammation and/or fibrosis of OA IFP are needed.

## Figures and Tables

**Table 1:** Infrapatellar fat pad histopathological scoring system.

IFP Histopathological Grading	ACL (*n* = 23)	End-Stage OA (*n* = 25)	*p*-Value
Lymphocytic Infiltration, number (%)	1 (3.6)	20 (80)	<0.0001
Grade 0, number (%)	22 (95.7)	5 (20)	
Grade 1, number (%)	1 (3.6)	7 (28)	
Grade 2, number (%)	0 (0)	13 (52)	
Vascularity, median (IQR)	8.9 (17.5–7.2)	30.6 (42.9–30.6)	<0.0001

ACL = anterior cruciate ligament; OA = osteoarthritis. Data are expressed as number (%) or median (IQR).

**Table 2:** Infrapatellar fat pad adipocytes characteristics.

IFP Adipocytes	ACL (*n* = 24)	End-Stage OA (*n* = 25)	*p*-Value
Area (μm^2^), median (IQR)	1798.03 (2362.46–1113.82)	3128.72 (3632.46–2583.45)	<0.0001
Major axis (μm), median (IQR)	53.60 (62.68–42.47)	72.16 (78.98–66.45)	<0.0001
Minor axis (μm), median (IQR)	39.79 (49.43–32.63)	52.73 (58.90–48.70)	<0.0001
eccentricity, median (IQR)	0.66 (0.70–0.63)	0.67 (0.70–0.64)	0.211

ACL = anterior cruciate ligament; OA = osteoarthritis. Data are expressed as median (IQR).

## Supplementary Materials

Supplementary materials can be found at https://www.mdpi.com/1422-0067/21/17/6016/s1.

## Abbreviations

ACLR	anterior cruciate ligament reconstruction
COLI	collagen Type I
COLIII	collagen Type Iii
COLVI	collagen Type Vi
CXCL8	C-X-C motif chemokine ligand 8
FABP4	fatty acid-binding protein 4
GPNMB	transmembrane glycoprotein NMB
HE	hematoxylin–eosin
IFP	infrapatellar fat pad
IL-6	interleukin-6
ITH5	inter-A-trypsin inhibitor heavy chain 5
MCP-1	monocyte chemotactic protein 1
OA	osteoarthritis
PPARγ	peroxisome proliferative activated receptor gamma
QRT-PCR	quantitative real-time PCR
SERPIN2	serine proteinase inhibitor 2
TGF-Β	transforming growth factor Β
TNF-A	tumor necrosis factor-A
TKR	total knee replacement
VEGF	vascular endothelial growth factor
