# Supplementary material for: Infrapatellar Fat Pad Gene Expression and Protein Production in Patients with and without Osteoarthritis
Source: Int J Mol Sci. 2020 Aug 21;21(17):6016. doi: 10.3390/ijms21176016 (PMC7503946; doi:10.3390/ijms21176016)
Supplement: Supplementary file 1 [file ijms-21-06016-s001.pdf]

International Journal of *Molecular Sciences*

MDPI

Supplementary materials

Table 1. Primer sets used for quantitative real-time PCR.

Gene	Forward Primer (5′–3′)	Reverse Primer (5′–3′)
**18 S**	CGGCTACCACATCCAAGGAA	GCTGGAATTACCGCGGCT
**TNF-α**	TGCTTGTTCCTCAGCCTCTT	GGGCTACAGGCTTGTCA
**IL-6**	TTCGGTACATCCTCGACG	AAGCATCCATCTTTTTCAGC
**MCP-1**	CCCCAGTCACCTGCTGTTAT	TGGAATCCTGAACCCACTTC
**PPARγ**	ACCCAGAAAGCGATTCCTTCA	AGTGGTCTTCCATTACGGAGAGATC
**Leptin**	GTGCGGATTCTTGTGGCTTT	GGAATGAAGTCCAAACCGGTG
**Adiponectin**	CCTGGTGAGAAGGGTGAGAA	CAATCCCACACTGAATGCTG
**FABP-4**	GAAAGTCAAGAGCACCATAACC	CCACCACCAGTTTATCATCC
**VEGF**	TCACCATGCAGATTATGCGGA	TGTTGTGCTGTAGGAAGCTCA
**COLI**	GAGAGCATGACCGATGG	GTGACGCTGTAGGTGAA
**COLIII**	GCAGGGTCTCCTGGTTCAAA	CGGGACCCATTTCGCCTTTA
**COLVI**	AAGGAGAACCTGGGAGGAAA	ACAGCGCTTCTCACAGTCG
**GPNMB**	CCCCTTCTTTAGGACCTGCTG	GTGATGGTGGCTTGAAAGTGG
**ITH5**	TCCTCAGGCTGGTCTCTGAT	GATGACTCTGCTCGGTGTGA
**SERPIN2**	CTTTGAGGATCCAGCCTCTG	TGCGTTTCTTTGTGTTCTCG

Table 2. Demographic data of ACL and end-stage OA patients.

	ACL Rupture	End-Stage OA	*p*
**Number of patients**	28	25	
**Sex, male number (%)**	21 (75%)	7 (28%)	0.001
**Age, years, median (IQR)**	31 (42-22)	68 (75-62)	<0.0001
**BMI, Kg/m², median (IQR)**	23.04 (25.26-20.57)	29.52 (32.25-25.95)	<0.0001

ACL = anterior cruciate ligament, OA = osteoarthritis, IQR = interquartile range, BMI = body mass index. Data are expressed as median (IQR).

Table 3. IFP immunohistochemistry grading.

IL-6 IHC Grading	ACL (*n* =21)	End-stage OA (*n* =14)	*p*
IL-6, number (%)	8 (38.1)	13 (92.9)	<0.001
Grade 0, number (%)	13 (46.4)	1 (4)	
Grade 1, number (%)	6 (21.4)	4 (16)	
Grade 2, number (%)	1 (3.6)	3 (12)	
Grade 3, number (%)	1 (3.6)	6 (24)	

IHC =immunohistochemistry, IFP = infrapatellar fat pad. Data are expressed as number (%).

Table 4. Generalized linear regression model to investigate the association of variables with BMI and age in all patients.

Response Variable	BMI (*p*-Value)	Age (*p*-Value)
**Vascularization**	0.744	0.922
**Lymphocytic infiltration**	0.310	0.734
**IL6 IHC Grade**	0.377	0.836
**Adipocyte Area**	0.033	0.385
**Number of adipocytes**	0.603	0.280

BMI = body max index, IHC = immunohistochemistry.

Table 5. Generalized linear regression model to investigate the association of variables with BMI and age in ACL group.

Response Variable	BMI (p-Value)	Age (p-Value)
**Vascularization**	0.510	0.668
**Lymphocytic infiltration**	0.518	0.470
**IL6 IHC Grade**	0.286	0.613
**Adipocyte Area**	0.477	0.125
**Number of adipocytes**	0.796	0.207

BMI = body max index, IHC = immunohistochemistry, ACL = anterior cruciate ligament.

Table 6. Generalized linear regression model to investigate the association of variables with BMI and age in End-stage OA group.

Response Variable	BMI (p-Value)	Age (p-Value)
**Vascularization**	0.468	0.662
**Lymphocytic infiltration**	0.475	0.836
**IL6 IHC Grade**	0.811	0.070
**Adipocyte Area**	0.057	0.329
**Number of adipocytes**	0.235	0.410

BMI = body max index, IHC = immunohistochemistry, OA = osteoarthritis.

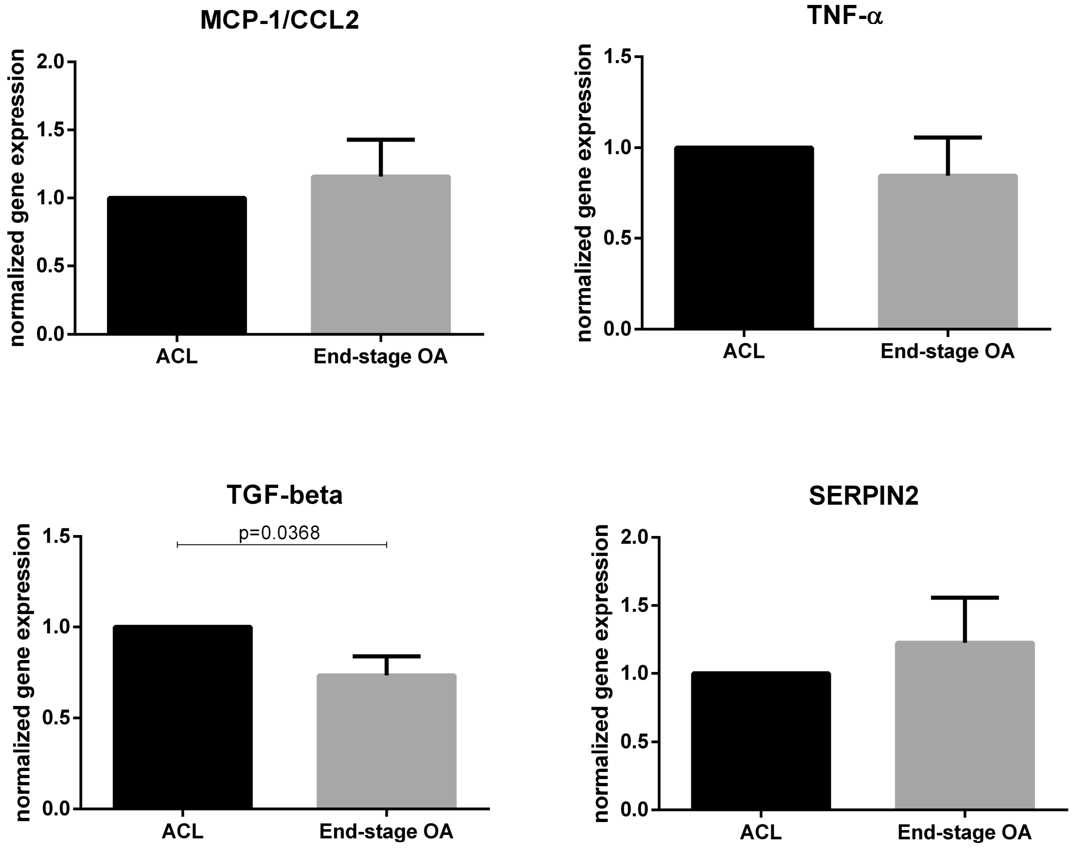

Figure 1 Monocyte Chemoattractant Protein-1 (MCP-1), Tumor necrosis factor (TNF-α), Transforming growth factor beta (TGF-beta) and serine proteinase inhibitor 2 (SERPIN 2) gene expression levels in ACL (n = 8) and end-stage OA IFP (n = 8). ACL = anterior cruciate ligament, OA = osteoarthritis.

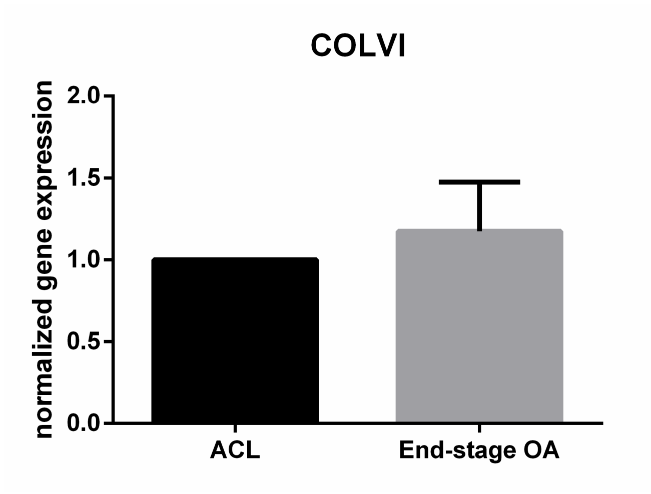

Figure 2. Collagen VI gene expression levels in ACL (n = 8) and end-stage OA IFP (n = 8). ACL = anterior cruciate ligament, OA = osteoarthritis.

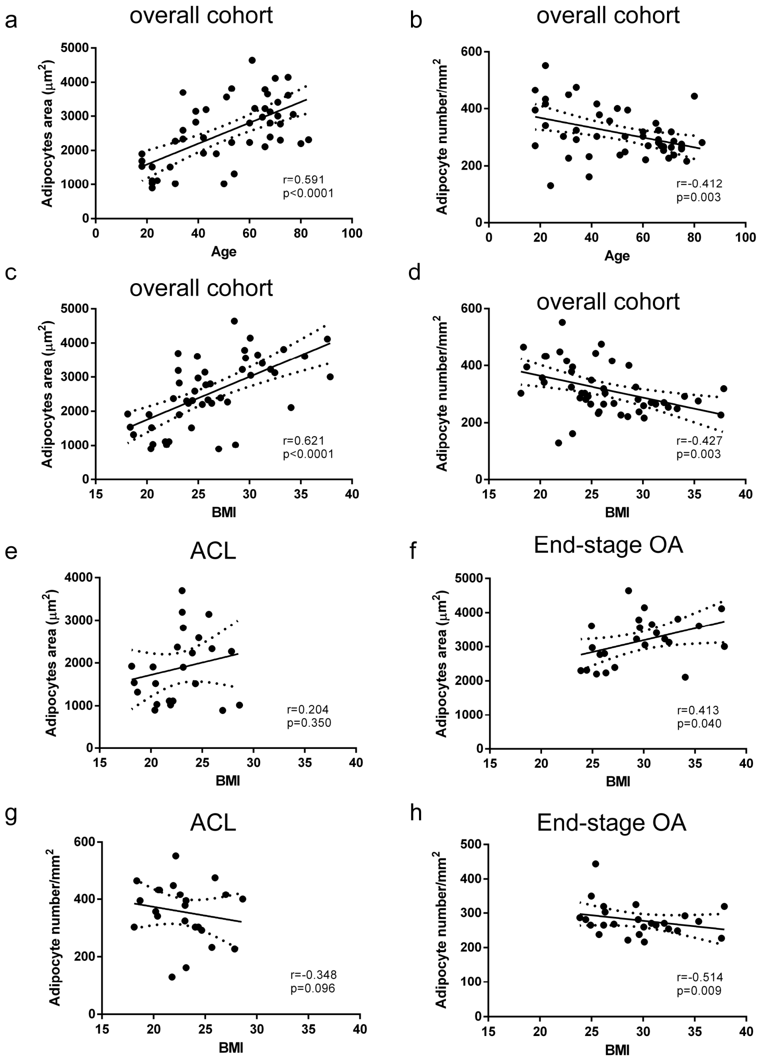

Figure 3. Correlations between adipocyte area, adipocyte number, age and BMI.
